# Editorial: Omics Sciences in microbiology and infectious diseases

**DOI:** 10.3389/fcimb.2026.1928147

**Published:** 2026-07-15

**Authors:** Andrea Marino, Stefano Marletta, Stefano Stracquadanio

**Affiliations:** 1 Department of Clinical and Experimental Medicine, Unit of Infectious Diseases, Azienda Ospedaliera di Rilievo Nazionale e di Alta Specializzazione (ARNAS) Garibaldi Hospital, University of Catania, Catania, Italy; 2 Department of Biomedical Science, Humanitas University, Milan, Italy; 3 Division of Pathology, Humanitas Istituto Clinico Catanese, Catania, Italy; 4 Department of Medicine and Surgery, “Kore” University of Enna, Enna, Italy

**Keywords:** antimicrobial resistance, clinical microbiology, epidemiology, infectious diseases, molecular analyses, omic sciences

The history of clinical microbiology is inseparable from the history of technological innovation. Every major methodological advance—from microscopy to culture-based techniques, from biochemical testing to molecular diagnostics—has not only improved our ability to identify infectious agents but has also expanded the questions microbiologists are able to address. The advent of high-throughput molecular technologies represents the latest and perhaps most profound step in this evolution (Didelot et al., 2012). Rather than simply increasing analytical performance, genomics, metagenomics, transcriptomics, proteomics, glycomics, and advanced bioinformatic tools have fundamentally redefined how infectious diseases are investigated. Today, microorganisms are no longer examined as isolated entities but as dynamic components of complex biological systems in which microbial evolution, host responses, environmental pressures, and ecological interactions continuously shape the course of infection (Hasin et al., 2017; Khan et al., 2019).

This conceptual shift extends well beyond technological innovation. Infectious diseases are increasingly understood as the outcome of interconnected molecular events rather than the consequence of a single pathogen acting in isolation. Consequently, the objective of modern clinical microbiology is no longer limited to identifying the causative microorganism; it also encompasses understanding why pathogens emerge, how they adapt, how they interact with the host, and how they evolve under selective pressures imposed by antimicrobial therapy and changing ecosystems. By bringing together microbiology, molecular biology, computational sciences, epidemiology, and clinical medicine, integrated omics approaches have broadened the analytical horizon of the discipline and fostered a genuinely systems-oriented view of infection (Eckhardt et al., 2020).

Far from replacing conventional microbiology, these technologies have expanded its diagnostic and investigative capabilities. Culture, microscopy, phenotypic characterization, and susceptibility testing remain indispensable components of routine laboratory practice. Their integration with high-throughput molecular approaches provides a level of biological resolution that was previously unattainable, allowing laboratory findings to be interpreted within a broader functional and epidemiological context. Clinical microbiology is therefore evolving from a discipline focused on detecting pathogens into one dedicated to understanding the biological systems that drive infectious diseases (Didelot et al., 2012; Chiu and Miller, 2019).

This conceptual evolution is perhaps most evident in diagnostic microbiology. For decades, laboratory diagnosis relied primarily on culture-dependent techniques, targeted molecular assays, and phenotypic identification. Although these methods remain the cornerstone of routine practice, they are often constrained by lengthy turnaround times, limited sensitivity, or the inability to detect fastidious, uncultivable, or unexpected microorganisms. The introduction of next-generation sequencing has profoundly expanded these diagnostic possibilities by enabling comprehensive molecular characterization directly from clinical specimens (Mitchell and Simner, 2019).

The studies by Ni et al., Zhang et al., Song et al., Slunečko et al., and Gatti et al., included in this Research Topic further illustrate the broad applicability of targeted sequencing, long-read sequencing, and clinical metagenomics across a wide range of bacterial and parasitic infections, demonstrating their value for the diagnosis of both common and diagnostically challenging pathogens. Unlike conventional diagnostic workflows, these approaches allow the simultaneous detection of multiple microorganisms, reveal strain-level diversity, and identify clinically relevant genetic determinants associated with virulence or antimicrobial resistance. For example, the contribution by Zhang et al. describes the successful integration of LAMP amplification with CRISPR/Cas12b detection for rapid identification of *Helicobacter pylori*, illustrating how innovative molecular platforms can substantially shorten diagnostic turnaround while maintaining high analytical performance.

Importantly, the diagnostic contribution of modern molecular profiling extends far beyond genome sequencing. Among the contributions in this Research Topic, Slunečko et al. and Lee et al. demonstrate that metagenomic approaches provide comprehensive characterization of complex microbial communities and polymicrobial infections, whereas transcriptomics and proteomics offer functional information by revealing which microbial pathways are actively engaged during infection. Similarly, Dadovska et al. highlights the expanding contribution of glycomic analyses through the characterization of microbial polysaccharides as novel structural fingerprints with potential diagnostic applications. Together, these complementary technologies generate a multidimensional representation of infection that cannot be achieved through any individual analytical platform.

The role of diagnostic microbiology is therefore changing. Rather than representing the final outcome of laboratory investigation, pathogen identification has become the starting point for a broader process of molecular interpretation that integrates microbial characterization, antimicrobial resistance profiling, epidemiological investigation, and increasingly, prediction of disease evolution and therapeutic response (Didelot et al., 2012; Chiu and Miller, 2019). Diagnosis is no longer the endpoint of microbiology; it has become the entry point to precision microbiology.

Although improved diagnosis has been one of the earliest clinical applications of omics technologies, their contribution extends far beyond pathogen identification. Equally important is their ability to explain the biological mechanisms that govern microbial behavior and host–pathogen interactions (Hasin et al., 2017; Khan et al., 2019). Genomics reveals the genetic repertoire of microorganisms; transcriptomics captures the dynamic regulation of gene expression; proteomics identifies the functional molecules responsible for cellular activities; and glycomics uncovers the molecular interfaces through which pathogens recognize host tissues, evade immune responses, and establish infection. Individually, each platform offers only a partial perspective. Viewed collectively, these complementary approaches shift the focus from describing microbial components to understanding the biological processes that determine infection. Together, they provide a comprehensive framework for understanding the molecular complexity of infectious diseases (Hasin et al., 2017).

The studies included in this Research Topic further reinforce this concept. Zheng et al. employs comparative genomics to investigate the genetic basis of microbial evolution, adaptation, and virulence, illustrating how high-resolution genomic analyses can identify determinants associated with pathogenicity and ecological specialization. Yet microbial genomes describe biological potential rather than biological activity. Integrating transcriptomic and proteomic data has revealed sophisticated regulatory networks that enable pathogens to survive environmental stress, modulate virulence programs, and persist within hostile host environments. This concept is exemplified by Grigorov et al., which combines transcriptomic and proteomic profiling to characterize the adaptive response of *Mycobacterium abscessus* to prolonged potassium deficiency and starvation. Likewise, detailed molecular characterization of virulence-associated factors has clarified how specific microbial components influence host immune responses and disease progression. In this regard, the investigation of virulence-associated proteins in *Streptococcus mutans* by Matsuoka et al. provides an excellent example of how proteomic analyses can uncover molecular mechanisms underlying bacterial pathogenicity. Collectively, these contributions demonstrate that pathogenicity emerges from coordinated molecular processes rather than from isolated genes or individual proteins.

Perhaps the most significant conceptual advance introduced by multi-omics is the transition from studying pathogens in isolation to investigating infection as a dynamic biological system. Combining microbial and host-derived molecular information is progressively revealing biomarkers associated with disease severity, identifying mechanisms of immune dysregulation, and generating novel hypotheses for therapeutic intervention. Such a systems-level perspective also opens the way to more personalized approaches to infectious diseases, where both microbial characteristics and host biology contribute to clinical decision-making. An illustrative example is the study by 12, which highlights the potential of microbiome profiling to predict therapeutic outcomes and guide personalized treatment strategies.

Understanding infection today requires much more than identifying microorganisms or cataloguing their genomes. The real strength of integrated omics lies in its ability to connect genes, transcripts, proteins, glycans, microbial communities, and host responses into coherent biological models that explain why infections develop, evolve, and ultimately differ from one patient to another (Hasin et al., 2017; Khan et al., 2019; Stracquadanio et al., 2024).

The same systems-level perspective also extends beyond the individual patient. Molecular information is increasingly becoming epidemiological information, expanding the role of clinical microbiology from laboratory diagnosis to population health (Chiu and Miller, 2019). In this context, whole-genome sequencing has become one of the most powerful tools for reconstructing transmission pathways, monitoring antimicrobial resistance, identifying emerging lineages, and supporting real-time surveillance. Rather than replacing traditional epidemiological methods, genomic analyses have complemented them by providing an unprecedented level of biological resolution that links individual clinical isolates to broader patterns of pathogen evolution and dissemination (Didelot et al., 2012).

This systems-oriented perspective is particularly evident in studies addressing bacterial taxonomy and antimicrobial resistance. High-resolution genomic analyses have substantially refined species identification, especially for uncommon or taxonomically challenging microorganisms that frequently escape conventional diagnostic algorithms. This concept is exemplified by the identification described by Gatti et al. of a diagnostically unresolved case of *Mycobacterium saskatchewanense* through an NGS-based approach, highlighting the value of genomics for resolving uncommon or taxonomically challenging pathogens. At the same time, complete genome sequencing has considerably improved our understanding of resistance evolution by revealing the organization, mobility, and dissemination of plasmids and other mobile genetic elements carrying antimicrobial resistance determinants. Likewise, Wang et al. describes a multidrug-resistant *Pseudomonas fulva* isolate carrying a transferable megaplasmid harboring multiple resistance determinants, illustrating the ecological complexity underlying antimicrobial resistance dissemination. These findings demonstrate that resistance should no longer be regarded simply as a characteristic of individual pathogens but as a dynamic ecological process driven by microbial evolution, horizontal gene transfer, and environmental selective pressures.

Large-scale genomic surveillance further illustrates how molecular data can be translated into actionable epidemiological knowledge. Korneenko et al. provides a compelling example of genomic surveillance by documenting the epidemiology of a recent *Mycoplasma pneumoniae* outbreak and revealing regional differences in circulating strains and antimicrobial resistance profiles. Such information is becoming increasingly valuable for infection prevention, antimicrobial stewardship, and public health preparedness. As sequencing technologies continue to integrate into routine microbiological practice, the historical distinction between clinical diagnostics and epidemiological surveillance is becoming progressively less defined.

Clinical microbiology is therefore extending beyond the laboratory bench. By connecting molecular findings with epidemiological evidence, integrated omics approaches are creating a continuum that links individual patients to populations, transforming biological data into informed public health action (Didelot et al., 2012).

Despite these remarkable advances, important challenges remain before the full potential of integrated molecular approaches can be translated into routine clinical practice. Generating large-scale datasets is no longer the principal obstacle. The challenge is no longer generating molecular data, but extracting biologically meaningful knowledge from increasingly complex datasets through their integration and clinical interpretation (Khan et al., 2019). Genomic, transcriptomic, proteomic, glycomic, and metagenomic datasets each capture different dimensions of infection; only their integration can provide a comprehensive understanding of disease biology (Hasin et al., 2017; Stracquadanio et al., 2024).

Bioinformatics has consequently evolved from a supporting discipline into a central component of modern microbiology. Robust computational pipelines, standardized analytical workflows, harmonized reporting criteria, and interoperable databases are becoming indispensable for ensuring that molecular information is reproducible, comparable, and clinically interpretable. At the same time, advances in artificial intelligence and machine learning are enhancing both molecular data interpretation and diagnostic workflows by facilitating the analysis of complex datasets, improving microorganism detection, identifying hidden biological relationships, predicting antimicrobial resistance, and supporting clinical decision-making (Marletta et al., 2023; Gador-Whyte et al., 2026). These computational tools should not be viewed as alternatives to microbiological expertise but as complementary resources capable of enhancing biological interpretation.

Technological innovation should not obscure the enduring importance of conventional microbiology. Culture, microscopy, phenotypic antimicrobial susceptibility testing, and careful clinical interpretation remain indispensable for patient management. The future of infectious disease research will therefore depend not on replacing traditional microbiology, but on integrating established laboratory methods with complementary molecular technologies into a unified diagnostic and investigative framework (Chiu and Miller, 2019).

Ultimately, the next generation of clinical microbiology will be defined not by the quantity of molecular data that laboratories are able to generate, but by their ability to integrate those data into meaningful biological knowledge that improves diagnosis, guides therapy, and informs public health.

Looking across the studies collected in this Research Topic, one message emerges with remarkable clarity. Omics sciences are no longer defined by individual technologies, but by their capacity to integrate complementary layers of biological information into a unified understanding of infectious diseases. The articles included in this Research Topic span an exceptional diversity of pathogens, analytical platforms, and clinical applications. Yet, despite this diversity, they collectively convey a single message: integrating complementary omics approaches provides a deeper and more clinically meaningful understanding of infectious diseases than any individual technology alone.

Collectively, these studies illustrate the remarkable breadth of contemporary microbiology. They range from innovative diagnostic platforms and comparative genomics to metagenomic analyses, glycomic investigations, antimicrobial resistance, pathogen evolution, and genomic surveillance. Considered individually, each contribution advances knowledge within its own field; viewed together, they provide a broader perspective on how modern microbiology is evolving toward an increasingly integrated and systems-oriented discipline. This diversity reflects one of the greatest strengths of contemporary omics research: no single technology can fully explain the complexity of infection, whereas complementary approaches provide insights that are both biologically richer and clinically more informative.

As Guest Editors, we hope that this Research Topic will encourage further collaboration across disciplines and stimulate the continued integration of molecular, computational, microbiological, and clinical expertise (Eckhardt et al., 2020). The future of infectious disease research will not be shaped by genomics, proteomics, transcriptomics, metagenomics, or glycomics considered independently, but by their convergence into a coherent framework capable of translating molecular complexity into biological understanding and clinical benefit.

Clinical microbiology is evolving into systems microbiology. The true promise of omics sciences lies not in generating more molecular data, but in enabling a deeper understanding of infectious diseases that ultimately benefits patients, healthcare systems, and public health.
